# Predicting Helical Topologies in RNA Junctions as Tree Graphs

**DOI:** 10.1371/journal.pone.0071947

**Published:** 2013-08-26

**Authors:** Christian Laing, Segun Jung, Namhee Kim, Shereef Elmetwaly, Mai Zahran, Tamar Schlick

**Affiliations:** 1 Department of Biology, Wilkes University, Wilkes-Barre, Pennsylvania, United States of America; 2 Department of Mathematics and Computer Science, Wilkes University, Wilkes-Barre, Pennsylvania, United States of America; 3 Department of Chemistry, New York University, New York, United States of America; 4 Courant Institute of Mathematical Sciences, New York University, New York, United States of America; Universite de Sherbrooke, Canada

## Abstract

RNA molecules are important cellular components involved in many fundamental biological processes. Understanding the mechanisms behind their functions requires knowledge of their tertiary structures. Though computational RNA folding approaches exist, they often require manual manipulation and expert intuition; predicting global long-range tertiary contacts remains challenging. Here we develop a computational approach and associated program module (RNAJAG) to predict helical arrangements/topologies in RNA junctions. Our method has two components: junction topology prediction and graph modeling. First, junction topologies are determined by a data mining approach from a given secondary structure of the target RNAs; second, the predicted topology is used to construct a tree graph consistent with geometric preferences analyzed from solved RNAs. The predicted graphs, which model the helical arrangements of RNA junctions for a large set of 200 junctions using a cross validation procedure, yield fairly good representations compared to the helical configurations in native RNAs, and can be further used to develop all-atom models as we show for two examples. Because junctions are among the most complex structural elements in RNA, this work advances folding structure prediction methods of large RNAs. The RNAJAG module is available to academic users upon request.

## Introduction

Exciting recent discoveries have made it clear that RNA functions much like a master programmer – far beyond information transfer and protein synthesis [1]–[4]. Indeed, RNA’s regulatory roles encompass RNA splicing, protein regulation, small-metabolite sensing, RNA interference, and RNA modifications among others. Intimately connected with these gene altering and editing roles are the structural properties of RNAs because they dictate the dynamics of RNAs as well as interactions with other molecules. The close connection between structure and function of RNAs is evident from the many recent studies of RNA tertiary motifs, as well as advances in various aspects of RNA structure; these advances have in turn stimulated efforts in the structure prediction of RNA (see [5] for de novo RNA structure prediction, and [6]–[10] for recent reviews on these topics of 3D structure modeling and prediction).

To aid in the study of RNA structure, mathematical and computational approaches have contributed to the RNA structure prediction field. For example, RNA2D3D [11] and ASSEMBLE [12] are semi-automated programs that build first-order approximations of RNA 3D models using secondary or tertiary structure information from homologous RNAs. Other automated 3D structure prediction programs have been developed; FARNA [13], iFoldRNA [14], and NAST [15] rely on coarse-grained modeling with simulations to fold RNAs with the guidance of physics or knowledge-based energy functions; MC-Sym [16] predicts all-atom models of RNA by inserting small cyclic motif fragments, collected from solved RNA structures. BARNACLE [17] uses a coarse-grained probabilistic model of RNA to predict atomic models by efficient sampling of RNA conformations. MOSAIC [18] is another approach to efficiently and accurately model RNAs by including the local and global hierarchical folding principles. While these advances are significant, current limitations of all such programs, however, lie in predicting large or complex RNA structures, mainly due to the large size of the conformational space. In particular, predicting the 3D structures of RNA junctions, formed by multiple helical arms, is challenging because the spatial organization is often determined by non-canonical base pairs and base stacking interactions occurring within the junction domain. Furthermore, even if these programs can successfully generate models that locally resemble native RNA structures, the spatial organization of helical elements in junctions tend to be inaccurate, thus requiring manual intervention, as recently reviewed by Laing and Schlick [6].

A reduction of the conformational space size can also be achieved with graphical representations of RNA. Indeed, using tree graphs to describe the discrete repertoire of RNA molecules has led to prediction of new RNA folds and design of novel motifs (see [8], [19] and Kim *et* *al.* submitted [20] for recent reviews). These graphical approaches began with pioneering works of Waterman [21], Shapiro [22], and others [23], [24]. Recently, we introduced the RNA-As-Graph (RAG) tree and dual graphs to represent RNA 2D topologies, catalogue all possible topologies [25], [26], and predict novel RNA motifs [26]–[29]. Knisley and coworkers applied the RAG tree graphs to analyze secondary structures of RNAs and predict larger RNA-like structures by merging two RNA graphs and applying neural network analysis [30]. Gopal *et* *al* applied RAG to model large viral RNAs [31]. Many other applications of RAG have been reported (see review in Kim *et* *al*. [19]). The much reduced RNA conformational space using these graphs opens new ways to describe and predict large RNA topologies.

Here we develop tree graph representations to model helical arrangements in RNA structures. We aim to efficiently sample the 3D conformational space and predict global orientations of RNA junctions, which are important structural elements that form when three or more helices come together in space. As input, we use knowledge of the secondary structure, which can be predicted from the sequence by using programs such as Mfold [32] and RNAfold [33] based on the dynamic programming algorithm first proposed by Nussinov [34], [35], or can be extracted from multiple sequence alignments [36] or from experimental techniques such as RNA probing [37], crystallography, and NMR (resources available in databases such as RNA STRAND [38] and Rfam [39]). The output is a graph model of the predicted junction topology.

**Figure 1:**
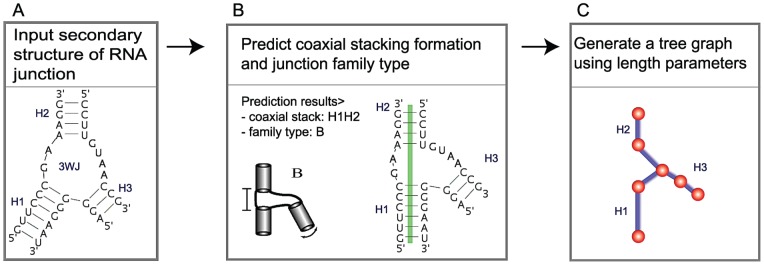
RNAJAG starts from an RNA secondary structure (A), uses Junction-Explorer to predict coaxial stacking and junction family types (B), and constructs a scaled tree graph using length parameters (C) .

Our new module denoted RNAJAG (RNA-Junction-As-Graph) predicts tree graphs of RNA junctions for a given secondary structure (see Figure 1 for the computational procedure). It expands upon our program Junction-Explorer in several important ways; first, RNAJAG generates a candidate junction graph model with specific helical arrangements (on top of family type/stacking orientation); second, the predicted graph incorporates native-like RNA junction features such as interhelical distances obtained from analysis of hundreds of solved RNA junction structures; third, the graph serves as basis to build all-atom models. Results show that RNAJAG reproduces native-like folds of helical arrangements in most junctions tested in the cross validation procedure (3- and 4-way junctions). Specifically, comparisons between our predicted tree graphs and the graphs obtained from solved crystal structures yield RMSD (root-mean-square deviation) values within range of 2–11Å (3-way) and 2–26Å (4-way), for all corresponding junctions. Importantly, the graph output of RNAJAG can be utilized to build coarse-grained or all-atom models and extend the approach to higher-order junctions. In addition, RNAJAG allows determining helical packing arrangements in junction domains (e.g., coaxial stacking) for larger RNAs, which is one of the main limitations among current RNA 3D prediction methods.

## Materials and Methods

### Overview

RNAJAG is the new module developed to represent RNA junctions as tree graphs and to generate a helical arrangement ensemble that approximates plausible 3D structures (Figure 1). This module combines our previous junction topology prediction program called Junction-Explorer [40] with models of scaled tree graphs. RNAJAG proceeds in two steps: first, an updated Junction-Explorer version (see below) determines the junction topology, as well as coaxial stacking patterns between helical elements of the target RNA junction; second, using the prediction results in the first step, a tree graph is constructed using scaling parameters to determine the length of every edge representing a helical axis as well as geometric parameters to position the edges in the junction domain. Details are provided below for these two steps followed by the analysis tools needed to assess our predicted graphs, namely converting crystal structures into graphs for measuring various geometric features and assessing the performance of RNAJAG.

### Junction topology prediction

**Figure 2:**
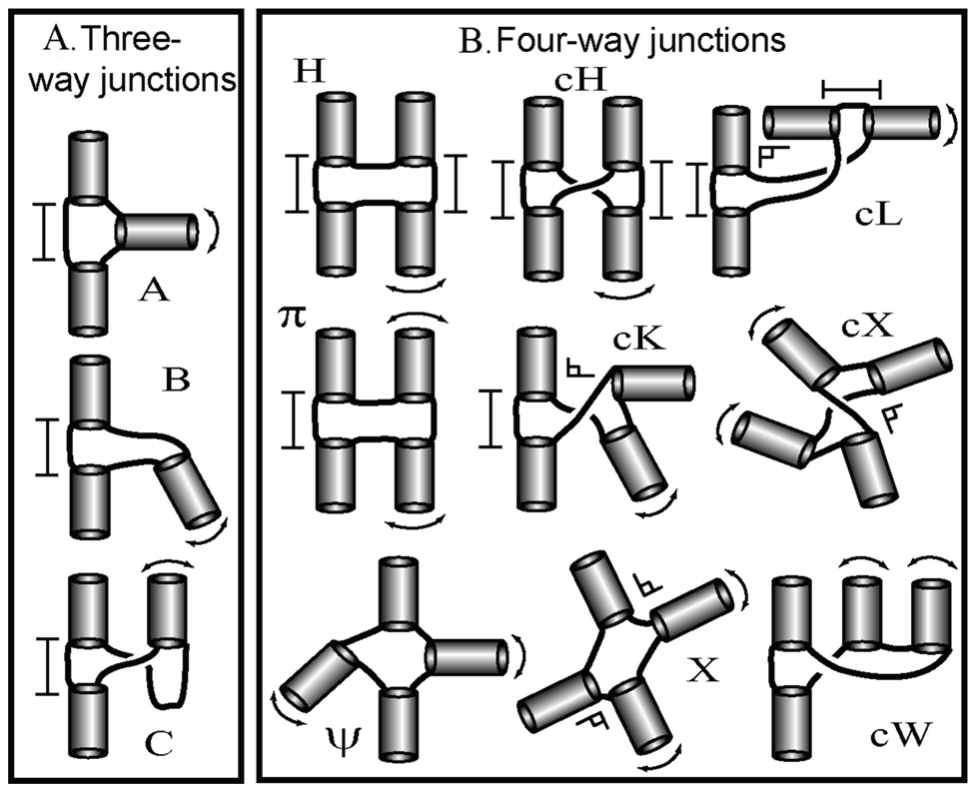
Schematic representation of 3- and 4-way junction families. (A) Three major family types – A, B, and C – are found in 3-way junctions where the helical arm, not involved in coaxial stacking, has different helical arrangements with respect to the coaxially stacked helices. (B) Nine major families – *H, cH, cL, cK, π, cW, Ψ, cX*, and *X* – are determined in 4-way junction based on coaxial stacking and overall helical arrangements.

Our analysis of RNA junction topologies [41], [42] is built upon previous topology analysis of 3-way junctions by Westhof and co-workers [43], who categorized three major families *A, B*, and *C* (Figure 2A). For 4-way junctions, we identified nine major families: *H, cH, cL, cK, 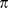, cW, 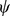, cX, and X* by coaxial stacking patterns and helical configurations (Figure 2B). Helices within RNA junctions prefer to arrange in parallel and perpendicular patterns, and conformations are stabilized using common 3D motifs like coaxial stacking, loop-helix interactions, and helix-packing interactions. Because the axes of helices in junctions tend to be coplanar [44], we represent junctions using planar tree graphs.

Junction-Explorer [40] uses a data mining approach known as random forests, which relies on multiple decision trees trained here using feature vectors (extracted from the 2D structures of solved RNAs used as the training dataset) for loop length, sequence, and other variables specified for any given junction; to determine the 2D information from the training dataset of 3D structures, we use three different programs – FR3D [45], MC-Annotate [46], and RNAVIEW [47] – and curate the 2D structures to contain only three base pairing types (AU, GC, or GU). We found some cases where programs yield different 2D structures; in such cases, we select the 2D structure with the lowest free energy among these programs as evaluated by the formation of AU, GC, or GU base pairs. To simplify the parsing of an RNA secondary structure into junctions, pseudoknots are automatically removed during the search. Similarly, because we aim to present a computational tool to predict helical arrangements within junctions based solely on a secondary structure, no knowledge from tertiary contacts (including pseudoknots) is introduced in an input secondary structure. Junction-Explorer uses these properties of RNA junctions as a function of sequence content and loop size to predict coaxial stacking patterns and junction family types. For example, a correct prediction of both the family type and coaxial stacking topology for the RNA in Figure 1B is family B and H_1_H_2_ stacking; family B with H_1_H_3_ stacking or family A with H_1_H_2_ would be incorrect in part.

Our updated version of Junction-Explorer uses an experimental dataset and a standard statistical analysis procedure. Our previous non-redundant junction dataset [40] was updated to include the most recent solved structures found in the PDB database as of October 2012. This dataset includes 130 3-way junctions, and 114 4-way junctions. With the exception of a few 3-way junctions with no coaxial stacks, most new junctions fit within the junction family classifications reported by the Westhof and Schlick groups [41], [43].

### Graph representation

**Figure 3:**
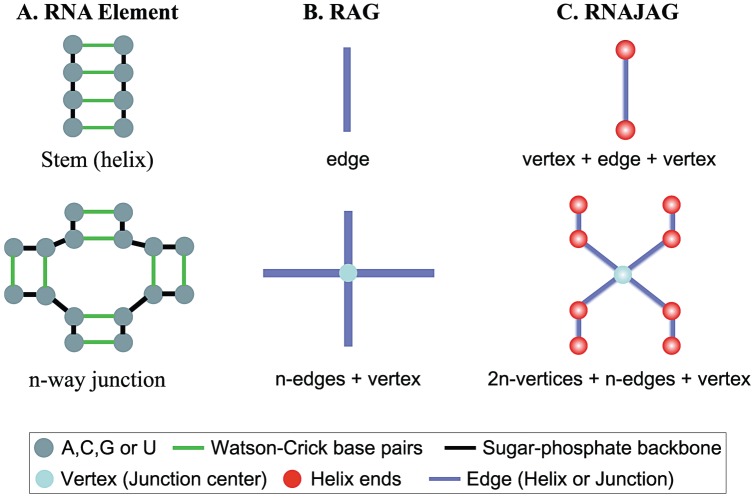
RNA graph representations. (A) RNA junction elements in a secondary structure. (B) RAG tree representation, which describes a helix as an edge and a loop as a vertex. (C) RNAJAG tree representation, which defines a helix using an edge and a loop and helix ends using a vertex.

Our previous graph theory work considered RNA-As-Graphs [48] to represent RNA secondary structures from a topological perspective [25], [49]. A RAG graph defines trees by representing helices as edges, and loop domains (hairpins, internal loops, and junctions) as vertices [50] (Figure 3A-B). This simple and intuitive representation provides the mathematical tools to estimate the RNA structural space as well as to predict yet unknown motifs [26].

In this work, we add further detail to the tree graphs to represent junction structures. We refine the RAG tree graphs by adding vertices at the terminal base pairs of a helix to represent helices of different lengths. We also include a vertex in the center of the junction domain to capture the junction’s spatial properties. In addition, we consider edges connecting the vertices at the end of helices, and edges to connect the end of a helix with the vertex in the center of the junction (Figure 3C). We illustrate how to translate RNA structures into RNA graphs, as well as the differences between RAG and RNAJAG, with two examples – a helix and a 4-way junction (Figure 3). This new graph representation captures properties of the helical organization for any degree of RNA junctions in 3D space.

### Translation of RNA crystal structures into graphs

**Figure 4:**
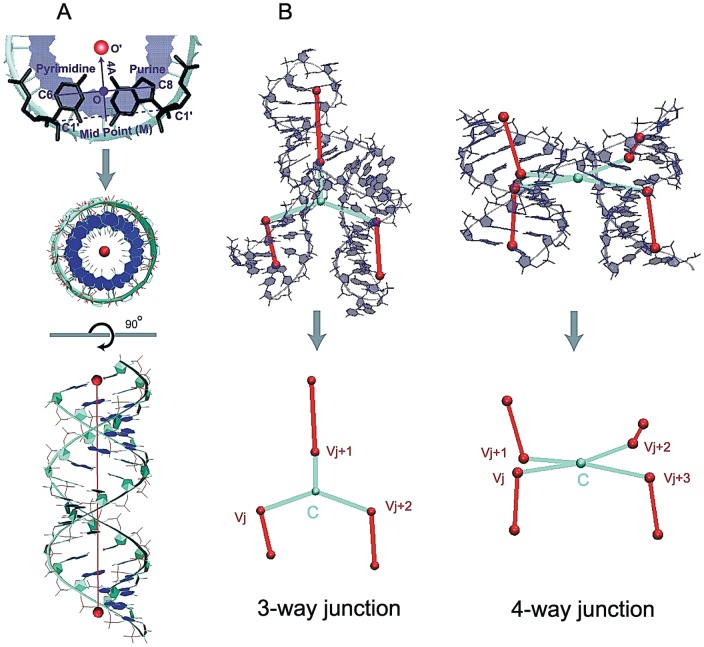
Graph representation of a helix and RNA junctions. (A) definition of coordinates for the origin (O′) of base pairs (see [51]) and a global helical axis for A-form RNA, from the top and the side. (B) Graphs of RNA junctions are obtained by translating helical branches into vertices and edges, and locating the center vertex *C* of each RNA junction (colored cyan); the center vertex *C* of an *n*-way junction is positioned as the average of adjacent vertices of *C* (*v_i_*, i = 1,…, *n, for n-way junction*) at helix ends.

To evaluate the accuracy of our approach for predicting helical arrangements via tree graphs, we generate a set of graphs obtained from solved crystal structures according to the definition of tree graphs described above. Thus, a helical element in an RNA junction is defined only if at least two consecutive Watson-Crick base pairs (GC and AU, and GU) are present. As described above, we represent each helix by two vertices and one edge: the vector origin (*O′*) of each vertex is determined by three steps: 1) find the midpoint *M* of C1′ atoms between the purine ((A)denine and (G)uanine) and pyrimidine ((C)ytosine and (U)racil) of the terminal base pairs of a helix; 2) consider the orthogonal projection from *M* to the line connecting the *C8* and *C6* atoms of the purine and pyrimidine, respectively; 3) scale the vector projection by 4Å as proposed by Schlick [51] (see Figure 4A). This definition for positioning a vertex is applied to both terminal base pairs of a helix. An edge is then added to connect the two adjacent vertices. Note that this edge aligns with the axis of the helix.

We extend this graph definition for helices to describe RNA junctions. For instance, an *n*-way junction translates into 2*n*+1 vertices – 2*n* vertices for *n* helices and one vertex for a junction centroid – and 2*n* edges; the junction centroid is an average of adjacent vertices *V_i_* (*i* = 1,…, *n*). Figure 4B illustrates examples of 3 and 4-way junctions and their translation into tree graphs; red edges represents helices while cyan edges illustrates the edges connecting the center of each junction to the helix edges. By converting a set of solved crystal structures into our graph notation, we can derive knowledge-based information about the spatial arrangements of helices within junctions. Table S1 shows the list of RNA junctions in solved crystal structures considered here.

### Distance parameter calculations using graphs

**Figure 5:**
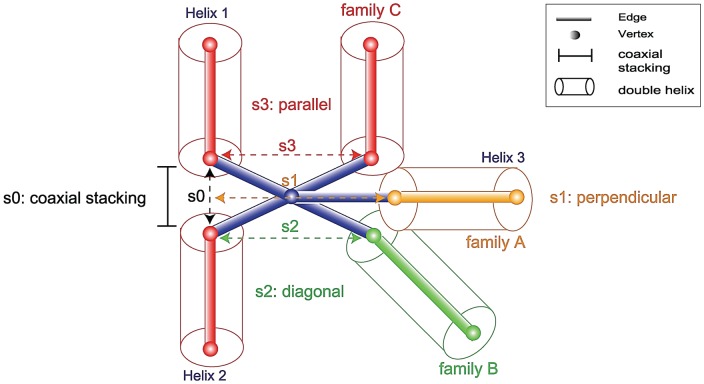
Scaling parameter calculations using graphs translated from crystal structures. The diagram shows the scaling distance parameter calculations for 3-way junctions where the scaling parameters s0, s1, s2, and s3 denote the distances between coaxial helices, perpendicular, diagonal, and parallel helical arrangements, respectively.

To determine the distance parameters to scale RNA graphs properly, we analyze structural data of 224 junctions collected from a non-redundant dataset of 47 solved crystal RNA structures (see Figure S1 and Table S1) and calculate the distances between coaxial helices, parallel, perpendicular, and diagonal helical arrangements in all 3 and 4-way junction elements of our graphs (Figure 5). We classify a ‘diagonal’ topology when the helix axis roughly forms a 45° angle with respect to the axis of stacked helices. Using linear regression we determine the distance between coaxial helical stacks by s0 = (2.75*L*+3.91)Å (R^2^  = 0.84), where L is the number of nucleotides between the helical elements forming coaxial stacks and R^2^ describes how well the linear regression fits the dataset (Figure S1A); the distances between parallel, perpendicular, and diagonal helical arrangements within junctions are determined by the position of unstacked helices with respect to the coaxially stacked helices (see s1, s2, and s3 in Figure 5 and S1) and reported as average values (with standard deviations) of 20.48(±5.25)Å, 19.95(±2.71)Å, and 21.17(±5.20)Å, respectively (see Figure S1 for the distance distributions).

In addition, we estimate the length of edge parameters representing the helical axis based on the distance of solved helical elements found within our non-redundant dataset. The helix length parameter is given by 2.87(*b*-1)Å, where *b* is the number of base pairs and 2.87Å corresponds to the base rise [51].

### Relation between graph and atomistic models

**Figure 6:**
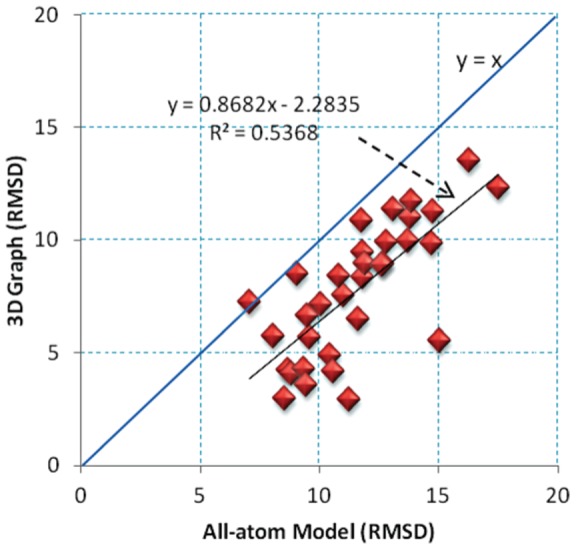
Statistical analysis of RMSDs for graphs with respect to their atomic models using a linear regression. Overall, a positive trend between all-atom models and graphs is observed with a slope value of 0.86.

To analyze the relation between root-mean-square deviation (RMSD) for tree graphs as opposed to atomic models, we calculate RMSDs for 13 all-atom models predicted from MC-Sym [16], NAST [15], and FARNA [13] against their corresponding all-atom native structures (33 calculations in total). This dataset of 13 structures composed of 3 or 4-way junctions was selected because both secondary and tertiary structures have been experimentally determined and they represent diverse features: the lengths vary from 51 to 117 nucleotides, and the topologies are diverse, including pseudoknots and loop-loop interactions. In addition, while some structures have been solved in the presence of proteins, others are structurally stable (e.g., tRNA), or rearrange upon binding to a substrate (e.g., ribozymes, riboswitches). We then build the tree graphs associated with these predicted atomistic models and compare these graphs to the corresponding graphs obtained from native structures (as described above). When performing a linear regression analysis using the RMSD values, we observe a positive correlation between all-atom and graph models (Figure 6). Thus, assessing graphs using the RMSD method is not equivalent to all-atom RMSD calculations but indicates similar trends.

### Comparing tree graphs using RMSD and MaxAngle calculations

We utilize two comparison methods – RMSD and Maximum Angle (MaxAngle) – to assess the quality of predicted graphs with respect to the native structures. The RMSD and MaxAngle [48] are useful for measuring global and local similarity of graphs, respectively.

The RMSD measures an average distance of vertices between superimposed graphs, defined as
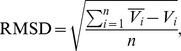
where *n* is the total number of vertices, and 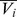 and 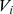 (1≤*i*≤*n*) are vertices in the reference and predicted graphs, respectively. To compare a pair of graphs, we translate these graphs into the origin, calculate an optimal rotation matrix using the singular value decomposition (program JAMA, adapted from a java matrix package (http://math.nist.gov/javanumerics/jama)), and superimpose them by a rotation matrix.

MaxAngle finds a maximum angle by calculating an angle of aligned two vectors of edges in the reference and predicted graphs defined by
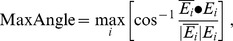
where *i* is the number of edges, and 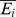 and 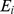 are vectors of edges from the reference and predicted graphs, respectively.

### Computational performance of RNAJAG and other RNA folding programs

To benchmark overall CPU times, we use the independent test dataset of 13 junctions described by Laing and Schlick [6] and determine CPU times for FARNA [13], MC-Sym [16], NAST [15], and RNAJAG.

We predict junction structures for MC-Sym from their own web server (http://www.major.iric.ca/MC-Sym/) and FARNA, NAST, and RNAJAG from our local machine, a 2×2.26 GHz Quad-Core Intel Xeon processor with 8GB of memory. Due to the different setup of each program, we cannot directly compare computational efficiency; however, the general trend is that to generate an RNA tree graph in RNAJAG (implemented in C++) takes less than 2 seconds CPU time; NAST is less CPU intensive than FARNA and MC-Sym. We found that all programs returned results in less than a few hours unless they failed to produce a model.

### Cross validation procedure

To evaluate the performance and the fitness level of our classifier, we perform the standard leave-one-out cross-validation (LOOCV) procedure [52] using feature vectors of 3-way and 4-way junctions. That is we train RNAJAG using the feature vectors collected from (*k–1*) junctions to test the remaining one.

We repeat this process for all (*k*) junctions, and report the predicted junction topology as well as RMSD between our predicted junction and its corresponding native structure represented by our graph. We use this procedure to show general performance on 200 RNA 3- and 4-way junctions (Table S2) as well as to demonstrate performance on 13 representatives RNA (Table 1).

**Table 1:** List of 13 RNA junctions from the PDB database.

PDB			Degree	Native Structure		RNAJAG			
	Nts	RNA Type		Coaxial Stacks	Family Type	Coaxial Stacks	Family Type	RMSD (Å)	Max Angle (°)
2FK6	52	tRNA	3WJ	H_1_H_3_	C	H_1_H_3_	A	4.01	166.43
1DK1	57	rRNA	3WJ	H_2_H_3_	A	H_2_H_3_	A	6.16	65.70
1MMS	58	rRNA	3WJ	H_1_H_3_	C	H_1_H_3_	C	4.13	16.36
3EGZ	65	Riboswitch	3WJ	H_2_H_3_	C	H_2_H_3_	C	6.59	46.63
2QUS	64	Ribozyme	3WJ	H_1_H_3_	C	H_1_H_2_	C	10.40	159.06
2OIU	51	Ribozyme	3WJ	H_1_H_2_	A	H_1_H_2_	A	2.12	28.98
3D2G	77	Riboswitch	3WJ	H_1_H_2_	A	H_1_H_2_	A	2.07	45.90
2HOJ	78	Riboswitch	3WJ	H_1_H_2_	A	H_1_H_2_	A	2.18	52.36
2GDI	80	Riboswitch	3WJ	H_1_H_2_	A	H_1_H_2_	A	1.98	58.95
1LNG	97	7S.S SRP	3WJ	H_1_H_3_	C	H_1_H_2_	A	9.04	62.26
1MFQ	117	7S.S SRP	3WJ	H_1_H_3_	C	H_1_H_3_	C	5.26	16.21
2DU3	71	tRNA	4WJ	H_1_H_4_,H_2_H_3_	cL	H_1_H_4_,H_2_H_3_	cL	2.01	29.51
2GIS	94	Riboswitch	4WJ	H_1_H_4_,H_2_H_3_	cL	H_1_H_4_,H_2_H_3_	cL	12.18	74.08

Each junction is listed with its junction family and coaxial stacking arrangement. RNAJAG achieves graphs with RMSD values below 11Å and 13Å for 3- and 4-way junctions, respectively.

### Building atomic models using graphs

Our general idea is to use a threading-like procedure to determine the atomic coordinates of the graphs predicted by RNAJAG based on a search for graph similarities in “3D-RAG”, an extension of the RAG database. 3D-RAG contains 3D atomic models extracted from high-resolution RNA crystal structures from the PDB databank; atomic structures are linked to corresponding 3D graphs. Figures S2-3 illustrate the build-up and search procedure of the 3D-RAG database (unpublished). The 3D graphs are classified based on RAG motif IDs, which reflect topological properties of secondary structural elements. We construct all-atom models in three steps (see Figure S3). First, we identify a motif ID of the target graph. Second, we compare the target graph to all 3D graphs catalogued with the same motif ID in 3D-RAG based on a standard RMSD calculation. Third, we select the graph with the lowest RMSD, extract its all-atom 3D coordinates, and verify that it contains the same number of nucleotides as the target sequence. The bases are then altered to match the target sequence as needed, while keeping the backbone intact.

If we do not find any structure match for the entire predicted RNAJAG graph, we partition the target graph into subgraphs and follow the procedure described above for each subgraph. We then assemble all the atomic fragments of the subgraphs to form a final all-atom RNA model. Energy minimization may be implemented in the future to relax the structure.

## Results

### RNAJAG prediction performance

**Figure 7:**
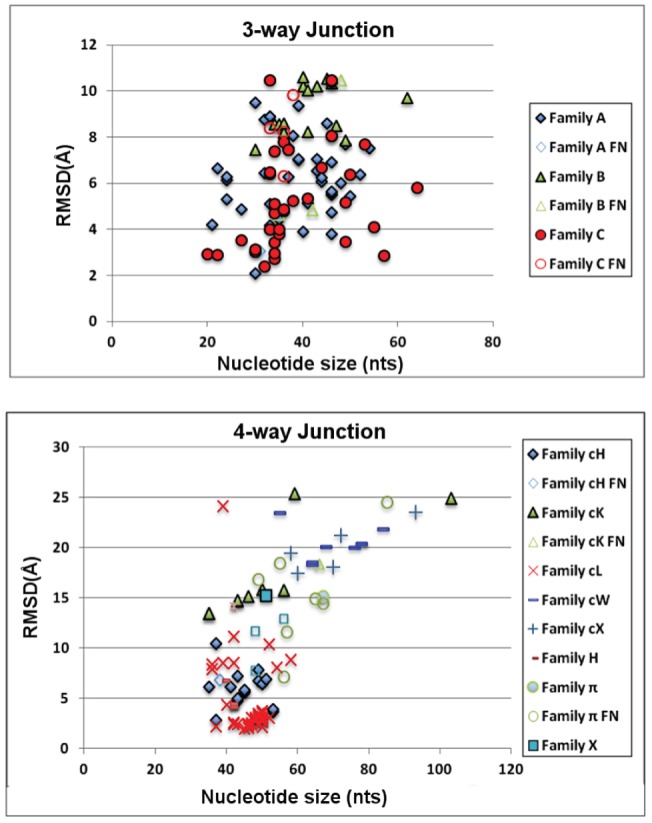
Distribution of RMSD scores for 3-way junctions (top) and 4-way junctions (bottom). The RMSD comparison is computed between the RNAJAG graphs and the graphs obtained from the PDB structures corresponding to the target RNA. Values are color-coded according to their correctly predicted family topology (solid colors), as well as the failed family predictions (false negatives with same shape but no filling).

To assess general RNAJAG performance, we consider the set of 200 junction domains (100 each for 3-way and 4-way systems) from high-resolution crystal structures as prediction targets. Results in Table S2 and Figure 7 (RMSD distributions) show that RNAJAG reproduces well native-like RNA folds in most of the 3- and 4-way junctions tested in the cross validation procedure. As the module RNAJAG consists of two components – junction topology prediction and graph modeling, we discuss the two parts in turn.

Overall, for the first component – junction topology – results indicate that the junction topology predictor module of RNAJAG, Junction-Explorer, identifies topologies and stacking patterns reasonably well for most of the test examples. Specifically, the module achieves accurate coaxial stacking prediction (95/100 for 3-way and 92/100 for 4-way) as well as junction family type (94/100 for 3-way and 87/100 for 4-way). Interestingly, most of the incorrect predictions for 4-way junctions correspond to families π and X, which are junction topologies rarely encountered. Other cases involving unusual inter or intra-molecular interactions (e.g., D-loop/T-loop interaction) are beyond the capability of our data mining approach and can lead to erroneous topology predictions.

**Figure 8:**
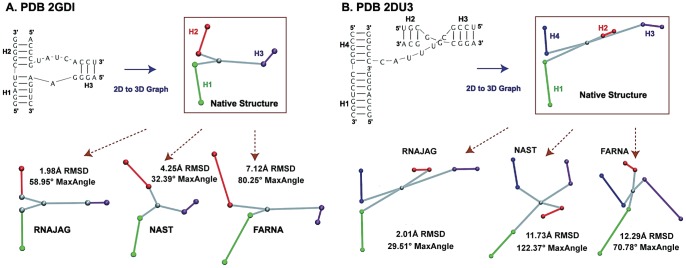
Prediction results of 3D modeling programs. Starting from the bottom left, a list of predictions from each program is presented by increasing RMSD values against the native structure in the counterclockwise direction. (A) 3-way junction of the TPP riboswitch (PDB 2GDI) with family type A and coaxial stacking between helices H_1_ and H_2_. Based on these examples, RNAJAG predicts most accurately followed by NAST, and FARNA. (B) 4-way junction of tRNA (PDB 2DU3) with family type cL and coaxial stacking between helices H_1_ and H_4_, and H_2_ and H_3_. After RNAJAG, NAST predicts most accurately followed by FARNA.

Our second component, graph modelling, builds a candidate model graph compatible with the predicted junction topology as described under Methods. These scaled tree graphs are generated and compared using RMSD and MaxAngle to those graphs from the corresponding native crystal structures. While RMSD is a global measure of graph similarity, MaxAngle, defined by a maximum angle of two aligned edge vectors (See Figure 8), is a local measure of accuracy that can help understand specific graph differences. For all 200 junctions considered, comparisons between our predicted and native tree graphs for all corresponding junctions yield RMSD values within range of 2–11Å (3-way) and 2–26Å (4-way). The RMSD values are presented and grouped by successful or missed junction family predictions in Figure 7. Interestingly, we note that for junctions corresponding to family C, our method produces reasonably graph junction models, while RMSDs for junctions belonging to family B perform poorly. A possible explanation is that for junction members of the family B, there is a high variability of the spatial arrangement between the coaxial stacking and its third helix. The parallel helical packing from junction elements of family C, on the other hand, tends to make a small variation because the coaxial stacking and its third helix often form long-range contacts. Similarly, we can observe that 4-way junction families of types cL, cH present better RMSD scores because these families are among the most abundant, and also present less variability in their inter-helical distances due to long-range contacts formed at the point of strand exchange.

We now analyze these RNAJAG results for a set of 13 representative RNAs of diverse sizes and functions (Table 1) by the same cross-validation procedure (leave-one-out). Correct junction topology classification is critical to achieve native-like graphs. Among the correct predictions for the junction topology in 3 and 4-way junctions are, for instance, the riboswitch (PDB 2GDI) and tRNA (PDB 2DU3), yielding best RMSD values of 1.98Å and 2.01Å, respectively.

**Figure 9:**
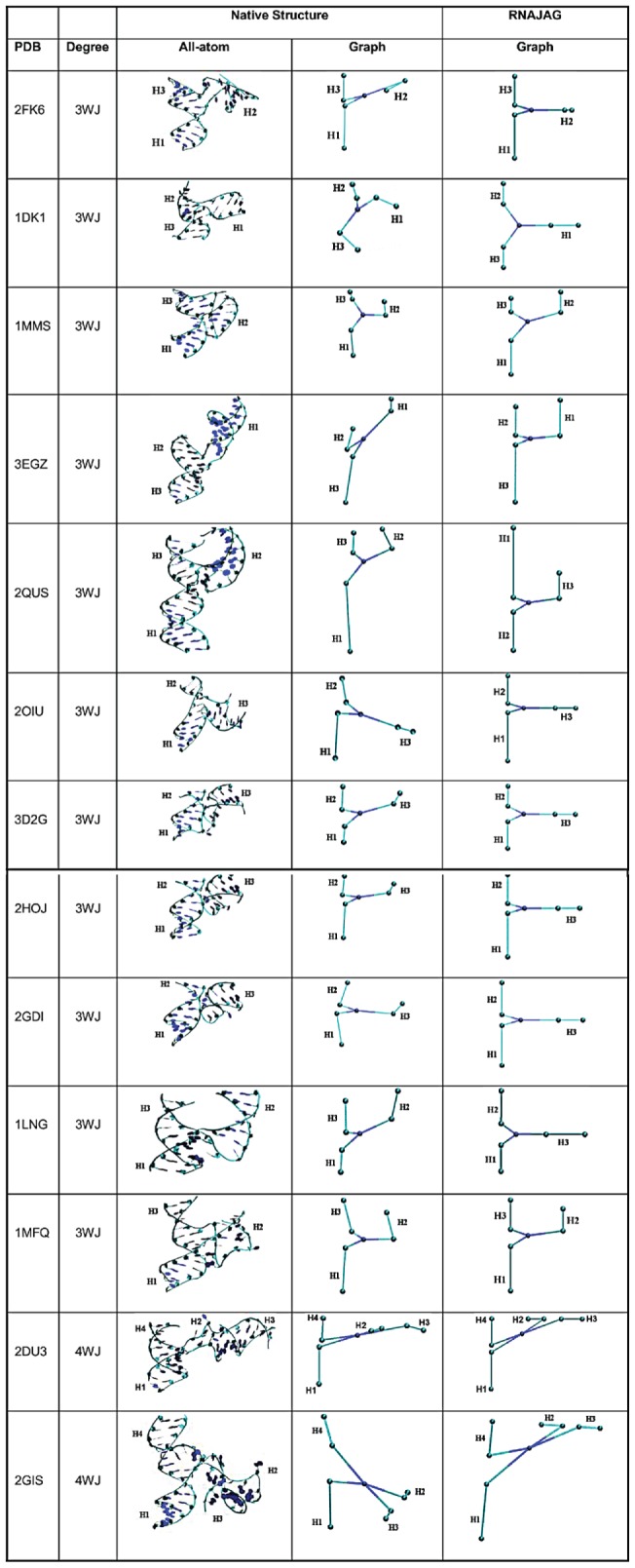
Graphs of the 13 RNA junctions. In each column from left to right, PDB entry, junction type, native structure, graph from native structure, and graph from RNAJAG are shown.

An example of a misclassification involves the tRNA (PDB 2FK6); it was assigned to a family A, but the native RNA structure forms a D-loop/T-loop motif (loop-loop tertiary interaction commonly observed in tRNA [53]) outside the junction domain that stabilizes its structural configuration as a family C (see Figure 9). Such misclassifications also occur for coaxial stacking; the hammerhead ribozyme (PDB 2QUS) was correctly classified in family type, but the coaxial stacking was predicted as H_1_H_2_ instead of H_1_H_3_. Finally, the signal recognition particle (PDB 1LNG) is incorrectly predicted, perhaps due to the small loop size differences, 1 *nt*, between H_1_H_2_ and H_1_H_3_ (see Figure 9).

Most RMSD values fall below 7Å except for the three examples (ribozyme (2QUS), SRP (1LNG), and riboswitch (2GIS)) that are within the range of 9 to 13Å. Similarly, most MaxAngle values fall below 75°, except for the two examples (tRNA (2KF6) and ribozyme (2QUS)) that have values higher than 159° due to incorrect topology predictions. The graphs (corresponding to RNAs listed in Table 1) are shown in Figure 9 for both the native structures and RNAJAG models.

### Comparison with other prediction methods

To compare the performance of RNAJAG with other programs, we made use of programs such as MC-Sym [16], NAST [15], and FARNA [13] to produce models from a selected set of 13 RNA junctions. The junctions consist of 3- and 4-way junctions and represent diverse features including nucleotide length and topology. To make comparisons at the graph level, we translate all predicted atomistic models into tree graphs using our graph definition (Figure 4, see details on ‘Materials and Methods’), and compute RMSD and MaxAngle against the corresponding graphs of native structures (graph from predicted structure vs. graph from crystal structure). The results are presented in Table 2 and the distributions in Figure S4.

**Table 2:** Comparison between RNAJAG and other tertiary structure prediction programs.

	RMSD (Å)				MaxAngle (°)			
PDB	RNAJAG	MC-Sym	NAST	FARNA	RNAJAG	MC-Sym	NAST	FARNA
2FK6	**4.01**	8.51	N/A	11.38	166.43	108.31	N/A	**49.10**
1DK1	6.16	5.74	**4.06**	6.63	65.7	129.42	**46.19**	140.39
1MMS	4.13	9.85	**2.89**	9.46	**16.36**	32.46	96.15	112.55
3EGZ	6.59	**5.53**	5.69	9.90	46.63	50.32	**36.04**	72.07
2QUS	10.40	**8.34**	10.86	8.94	159.06	130.49	59.80	**42.45**
2OIU	**2.12**	4.21	N/A	8.39	**28.98**	71.55	N/A	84.00
3D2G	**2.07**	N/A	N/A	3.56	**45.90**	N/A	N/A	88.84
2HOJ	**2.18**	N/A	N/A	4.17	**52.36**	N/A	N/A	74.07
2GDI	**1.98**	N/A	4.25	7.12	58.95	N/A	**32.39**	80.25
1LNG	9.04	**6.47**	7.55	8.92	62.26	80.16	**57.62**	73.84
1MFQ	**5.26**	9.93	11.01	8.88	**16.21**	82.10	83.05	78.30
2DU3	**2.01**	N/A	11.73	12.29	**29.51**	N/A	122.37	70.78
2GIS	12.18	13.51	N/A	**11.27**	**74.08**	101.37	N/A	122.67

Only the junction domain is considered for the RMSD and MaxAngle calculation using graph representation. The best RMSD and MaxAngle values for each structure are highlighted in bold on background. We denote N/A for those structures that other programs failed to predict using secondary structure information.

Although comparative RMSD values with respect to graphs and atomic models are not interchangeable, they are closely correlated as discussed in ‘Materials and Methods.’ Our statistical analysis uncovers the relationship between atomic models and their translated graphs, indicating that atomic models are well described in highly coarse-grained models (Figure 6).

We observe that both the RMSD and MaxAngle values range widely depending on the program. Specifically, RNAJAG produces a wider range of RMSD values varying from 1.9–12.2Å, with the largest values occurring mostly when coaxial helices or junction family (or both) are inaccurately predicted. In tandem, the best prediction values are observed when RNAJAG correctly classifies both the junction family type and coaxial stacking formation. The RMSD values for MC-Sym range from 4.2–13.5Å, NAST from 2.9–11.7Å, and FARNA from 3.5–12.3Å. By considering the number of predicted structures with best RMSDs over these 13 test cases, RNAJAG outperforms with 7 predictions followed by MC-Sym, NAST, and FARNA for 3 or less. MC-Sym and NAST often fail to predict structures, possibly due to some complications with the fragment insertion or assembly as reported in our previous study [6]. Although FARNA performs structure predictions least accurately, the program produces a model for all the structures along with RNAJAG.

To complement the RMSD measures, we also use MaxAngle to assess a local agreement of edges in the predicted graphs. The MaxAngle values for RNAJAG range from 16.2–166.4°, but mostly less than 65° with only three exceptions. Again, the largest (worst) values occur when RNAJAG fails to achieve the correct junction family and/or coaxial stacking patterns. The MaxAngle values for MC-Sym range from 32.4–130.5°, NAST from 32.4–122.4°, and FARNA from 42.4–140.4°. Overall, RNAJAG performs better on 7 of the 13 predictions, followed by NAST, FARNA, and MC-Sym for 4 or less.

Figure 8 presents two cases of graph comparisons between the native structure and graphs predicted by RNAJAG and the other programs to illustrate where predictions deviate from the experimental structure and from each other. The first example (Figure 8A) considers the 3-way junction structure of the TPP riboswitch (PDB 2GDI). When the RNAJAG graph is compared to the native one, RMSD and MaxAngle values of 1.98Å and 58.95°, respectively, are obtained. Interestingly, RNAJAG produces the best graph model with the lowest RMSD value, but not the lowest MaxAngle value; NAST yields a graph with the best MaxAngle value of 32.39°. Note that the graph conformations of RNAJAG for 3-way junctions are predefined by the major junction family types (Figure 2A) whereas NAST has much larger conformational space to explore, thus leading to a better fit of H_3_ to the native structure in this case. Our graph representation also gives ideal alignments for the coaxial helices, which is not always the case for graphs obtained from native structures, possibly due to helical rearrangements outside the junction domain.

The second case is the 4-way junction obtained from a Cys-tRNA transfer RNA (PDB 2DU3). In contrast with other programs, RNAJAG generates the typical L-shape with similar proportions to the native state (Figure 8B), without knowledge of the D-loop/T-loop interaction occurring outside the junction domain, and yields the lowest RMSD (2.01Å) and MaxAngle (29.51°) among the programs. Considering the RMSDs, NAST follows RNAJAG, with 11.73Å, and it is followed by FARNA (12.29Å). MC-Sym was unable to generate a model in these examples, possibly due to the insufficient number of cyclic motif fragments to insert.

In both prediction cases, RNAJAG configures most edges similar to the native structures; however, the scaling of the loop region in the tRNA (Figure 8B) is slightly inaccurate and would require additional information (e.g., tertiary motifs) for proper orientation.

### Building All-atom Models Using Graphs

Of course, predicted model graphs are only a starting point. Ultimately, a protocol to build atomic models is required. Using the threading/build-up procedure described in Methods, we illustrate this idea for two mid-sized (∼50 *nts*) junction structures (see Figure S2-3 for technical details).

**Figure 10:**
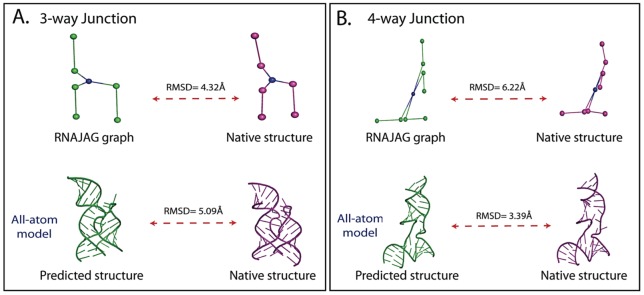
Derived all-atom models from predicted RNAJAG graphs using 3D-RAG threading for: (A) 3-way junction of a guanine-riboswitch RNA (PDB entry 3RKF) and (B) 4-way junction of a tRNA of Staphylococcus aureus (PDB entry 1QU2) .

The 3-way junction, guanine riboswitch RNA, is 53 *nts* long (PDB entry 3RKF) and belongs to the family type C. RNAJAG correctly predicts both the junction family type and the coaxial stacking and yields a graph with RMSD value of 4.32 Å with respect to the graph of its native structure (See Table 3 and Figure 10).

**Table 3:** All-atom modeling examples built from graphs.

PDB	Junction type	NTs	Graph (RMSD)	Atomic model (RMSD)
3RKF	3-way junction	53	4.32Å	5.09Å
1QU2	4-way junction	50	6.22Å	3.39Å

Both examples show comparable RMSD values (computed for all atoms except hydrogen) to the native structures.

We superimpose the predicted graph against all the graphs of the same motif ID family (namely (4, 2)) available in the 3D-RAG database, and order all these matches based on their RMSDs to the target graph. We extract the all-atom coordinates of the lowest RMSD graph (4.41Å), and create a model by mutating the bases to match the query sequence. We obtain an RMSD value of 5.09Å for the all-atom model junction region compared to its native structure.

The 4-way junction topology of the tRNA of Staphylococcus aureus, 50 *nts* long (PDB entry 1QU2), is correctly predicted by RNAJAG. It generates graph with 6.22 Å RMSD compared to the graph of its native structure (See Table 3 and Figure 10).

Similar to the 3-way junction, we search the 3D-RAG database for graph similarities in the same motif ID family (5, 3). We verify the 2D structure and construct an atomic model by mutating the bases of the extracted structure to match the query sequence. We achieve an all-atom model with RMSD of 3.39Å against the junction native structure.

## Discussion

With the continuous discovery of novel RNAs, it is imperative to advance computational methods to determine RNA structure and thus help in understanding RNA function. A major limitation in the field of RNA structure is the size of RNA molecules that can be accurately predicted. Indeed, the structural complexity grows rapidly as molecular size increases.

RNA junctions are important structural components that are often difficult to determine at both the secondary and tertiary structure levels. To address this problem, we introduced here a new graph theoretic approach that is applied to model RNA junctions in 3D space. The simplicity of using tree graphs to represent RNA junctions allows us to sample the minimal conformational space, particularly on the assembly of helical elements. Although our tree graph notation cannot represent pseudoknots, the proximity in 3D space of edges representing helices in junctions can suggest the formation of long-range interactions (pseudoknots, kissing hairpins, loop-receptors, etc. [54]).

RNAJAG is the new module that predicts and builds helical models for RNA junctions as tree graphs and consists of two components – junction topology prediction and graph modeling. Using an updated version of Junction-Explorer [40], we determine both the junction family type and coaxial stacking patterns. Based on these prediction results, an RNA graph, consisting of vertices and edges, is then constructed using length parameters describing spatial arrangements of helices in junctions. Note that the accurate prediction performance of Junction-Explorer is a critical step in RNAJAG as the tree graph generation depends sensitively on the outcome of Junction-Explorer.

Overall, RNAJAG reproduces reliable helical arrangements of the junctions with competitive RMSD values, in the range of 2–11Å (3-way) and 2–26Å (4-way) (see Table S2). In addition, the predicted graphs described here are comparable or better than other RNA folding programs. Note that RMSDs for RNAs are generally much larger than scores from protein predictions [8], [55] and also have a larger volume per unit mass. Thus, while 6Å RMSD is generally considered poor for proteins, it is a good prediction for RNAs. For atomic models, other measures besides RMSDs have alternatively been proposed to better assess RNA predictions [55], [56]. This is partly because nucleotides have a larger molecular size than proteins (while the diameter of a α-helix is 12Å, a typical A-DNA helix has a diameter of 23Å). The results from Table 2 show that our approach provides the largest number of best predictions, 7 for both RMSD and MaxAngle measures among compared graphs. Specifically, RNAJAG gives top 7 RMSD values compared to 3 or less out of 13 graphs with respect to MC-Sym, NAST and FARNA. Similarly, RNAJAG yields the top 7 MaxAngle measures compared to 4 or less for MC-Sym, NAST and FARNA.

Accurate predictions of Junction-Explorer in most instances make RNAJAG competitive with other programs. On the other hand, incorrect determinations of coaxial stacks and/or junction family types in a minority (20%) of the cases (Table 1) lead to dramatic deterioration of accuracy. The wide range of RMSD and MaxAngle values may reflect this possibility as reported in Table 2.

Our resulting tree graphs hold promise for further refinement of RNA structures. For example, our graphs can be used as starting templates to build coarse-grained or full atomic models using a threading/build-up procedure to link subgraph components and atomic structure (Figures S2-3). For these two examples, accurate all-atom models are achieved with RMSD values of 5.09Å and 3.39Å for 3 and 4-way junctions, respectively (Figure 10 and Table 3). Current work is focusing on generalizing this approach.

Although the tree graphs and all-atom models are not comparable, our statistical analysis shows that the RMSD measures of these two distinct models are positively correlated (Figure 6); a tree graph model is an oversimplified representation of the atomic RNA structure where helical elements and loop regions are mapped by a finite number of edges and vertices. Generally speaking, lower RMSD values for atomic models can be obtained compared to graph models. Additionally, we use MaxAngle to evaluate the quality of predicted local helical arrangements.

In this work we have primarily focused on pseudoknot-free 3 and 4-way junctions. These junctions represent over 80% of RNA junctions found in all available crystal structures to date [40]. RNAJAG can potentially be extended to predict higher order junctions since Junction-Explorer is capable of predicting coaxial stacking patterns for any junction order. For example, 5-way junctions can be partitioned into various possibilities of 3- and 4-way junctions [42], and thereby model the subset of junctions using RNAJAG.

Though our promising approach could be easily adapted to large RNAs with multiple junctions, several challenges remain with respect to the prediction accuracy of both the junction family and coaxial stacking configurations. For example, when loop-loop interaction motifs (e.g., PDB 2FK6) form outside the junction domain, they lead to unpredictable junction configurations. We also cannot account for protein-RNA interactions or solvent effects, challenges to all other tertiary structure prediction programs.

Finally, RNAJAG considers a limited range of the conformational space (Figure 2) [41], [43] since we only consider parallel, perpendicular, and diagonal helical arrangements. These orientations make graph generation very rapid; however, describing the dynamic nature of RNA structures requires flexible models, which can be addressed using coarse-grained or atomic models.

Additional ongoing work involves determining the optimal helical positions of the internal loops as well as the helical elements connecting these loops for large RNAs. Internal loops flanked by two helices can also be represented using tree graphs; therefore, preferred structural arrangements based on loop size and sequence content for them will improve the overall models. Ultimately, a pipeline that starts from our tree graphs and results in all-atom models can be envisioned. Combined with successful predictions of helices and internal loops, junction arrangement predictions could eventually provide a novel hierarchical approach to build tertiary RNA models for large RNA molecules.

## Supporting Information

Figure S1 **Distribution of distances with respect to various loop sizes for coaxial stacking of helices (A), parallel (B), perpendicular (C), and diagonal helical arrangements within junctions (D).**
(DOC)

Figure S2 **Illustration of the 3D-RAG build-up and search.** (A) All-atom structures extracted from known structures are translated into 3D graphs and partitioned into subgraphs based on RAG motif IDs. The subgraphs and all-atom fragments are catalogued in 3D-RAG. (B) 3D-RAG can be used for the search of graph similarity. After identifying the motif ID of the target graph, one can search for graph match in the motif ID selected, and extract the corresponding all-atom fragment.
(DOC)

Figure S3 **Illustration of the threading approach for the prediction of the all-atom RNA structure for a 3-way junction.** (A) Predicted graph by RNAJAG. (B) Search for graph similarities in 3D-RAG by superimposing the predicted RNAJAG graph with junction graphs of the same motif ID extracted from known structures. (C) Selection of the best graph candidate of known structures with the lowest RMSD, extraction of its all-atom coordinates from the database, and mutation of the bases to match those of the target sequence.
(DOC)

Figure S4 **Distribution of RMSD and MaxAngle for the representative 13 RNA junctions using RNAJAG and other 3D structure prediction programs.**
(DOC)

Table S1 **List of RNA 3D structures containing 224 junction data used for distance parameter estimation.**
(DOC)

Table S2 **List of 200 RNA junctions from the PDB database.** Each junction is listed with its junction family and coaxial stacking arrangement from the native structure and RNAJAG prediction. RNAJAG achieves graphs with RMSD values below 11Å and 26Å for 3- and 4-way junctions, respectively. Incorrect topology predictions are highlighted in bold.
(DOC)
