# Supplementary material for: Predicting Helical Topologies in RNA Junctions as Tree Graphs
Source: PLoS One. 2013 Aug 26;8(8):e71947. doi: 10.1371/journal.pone.0071947 (PMC3753280; doi:10.1371/journal.pone.0071947)
Supplement: Figure S1 — Distribution of distances with respect to various loop sizes for coaxial stacking of helices (A), parallel (B), perpendicular (C), and diagonal helical arrangements within junctions (D). (DOC) [file pone.0071947.s001.doc]

**Figure S1.** Distribution of distances with respect to various loop sizes for coaxial stacking of helices **(A)**, parallel **(B)**, perpendicular **(C)**, and diagonal helical arrangements within junctions **(D)**.
