# Supplementary material for: Predicting Helical Topologies in RNA Junctions as Tree Graphs
Source: PLoS One. 2013 Aug 26;8(8):e71947. doi: 10.1371/journal.pone.0071947 (PMC3753280; doi:10.1371/journal.pone.0071947)
Supplement: Figure S2 — Illustration of the 3D-RAG build-up and search. (A) All-atom structures extracted from known structures are translated into 3D graphs and partitioned into subgraphs based on RAG motif IDs. The subgraphs and all-atom fragments are catalogued in 3D-RAG. (B) 3D-RAG can be used for the search of graph similarity. After identifying the motif ID of the target graph, one can search for graph match in the motif ID selected, and extract the corresponding all-atom fragment. (DOC) [file pone.0071947.s002.doc]

**Figure S2**: Illustration of the 3D-RAG build-up and search. **(A)** All-atom structures extracted from known structures are translated into 3D graphs and partitioned into subgraphs based on RAG motif IDs. The subgraphs and all-atom fragments are catalogued in 3D-RAG. **(B)** 3D-RAG can be used for the search of graph similarity. After identifying the motif ID of the target graph, one can search for graph match in the motif ID selected, and extract the corresponding all-atom fragment.
