# Supplementary material for: Predicting Helical Topologies in RNA Junctions as Tree Graphs
Source: PLoS One. 2013 Aug 26;8(8):e71947. doi: 10.1371/journal.pone.0071947 (PMC3753280; doi:10.1371/journal.pone.0071947)
Supplement: Figure S3 — Illustration of the threading approach for the prediction of the all-atom RNA structure for a 3-way junction. (A) Predicted graph by RNAJAG. (B) Search for graph similarities in 3D-RAG by superimposing the predicted RNAJAG graph with junction graphs of the same motif ID extracted from known structures. (C) Selection of the best graph candidate of known structures with the lowest RMSD, extraction of its all-atom coordinates from the database, and mutation of the bases to match those of the target sequence. (DOC) [file pone.0071947.s003.doc]

**Figure S3**: Illustration of the threading approach for the prediction of the all-atom RNA structure for a 3-way junction. **(A)** Predicted graph by RNAJAG. **(B)** Search for graph similarities in 3D-RAG by superimposing the predicted RNAJAG graph with junction graphs of the same motif ID extracted from known structures. **(C)** Selection of the best graph candidate of known structures with the lowest RMSD, extraction of its all-atom coordinates from the database, and mutation of the bases to match those of the target sequence.
