# Supplementary material for: Predicting Helical Topologies in RNA Junctions as Tree Graphs
Source: PLoS One. 2013 Aug 26;8(8):e71947. doi: 10.1371/journal.pone.0071947 (PMC3753280; doi:10.1371/journal.pone.0071947)
Supplement: Figure S4 — Distribution of RMSD and MaxAngle for the representative 13 RNA junctions using RNAJAG and other 3D structure prediction programs. (DOC) [file pone.0071947.s004.doc]

**Figure S4**: Distribution of RMSD and MaxAngle for the representative 13 RNA junctions using RNAJAG and other 3D structure prediction programs.
