# Supplementary material for: Predicting Helical Topologies in RNA Junctions as Tree Graphs
Source: PLoS One. 2013 Aug 26;8(8):e71947. doi: 10.1371/journal.pone.0071947 (PMC3753280; doi:10.1371/journal.pone.0071947)
Supplement: Table S1 — List of RNA 3D structures containing 224 junction data used for distance parameter estimation. (DOC) [file pone.0071947.s005.doc]

**Table S1**. List of RNA 3D structures containing 224 junction data used for distance parameter estimation
