# Supplementary material for: Predicting Helical Topologies in RNA Junctions as Tree Graphs
Source: PLoS One. 2013 Aug 26;8(8):e71947. doi: 10.1371/journal.pone.0071947 (PMC3753280; doi:10.1371/journal.pone.0071947)
Supplement: Table S2 — List of 200 RNA junctions from the PDB database. Each junction is listed with its junction family and coaxial stacking arrangement from the native structure and RNAJAG prediction. RNAJAG achieves graphs with RMSD values below 11Å and 26Å for 3- and 4-way junctions, respectively. Incorrect topology predictions are highlighted in bold. (DOC) [file pone.0071947.s006.doc]

**Table S2**. List of 200 RNA junctions from the PDB database. Each junction is listed with its junction family and coaxial stacking arrangement from the native structure and RNAJAG prediction. RNAJAG achieves graphs with RMSD values below 11Å and 26Å for 3- and 4-way junctions, respectively. Incorrect topology predictions are highlighted in bold.
